# The Utilization of an Opioid-Free Anesthetic for Pediatric Circumcision in an Ambulatory Surgery Center

**DOI:** 10.3390/children8080678

**Published:** 2021-08-03

**Authors:** Laura E. Gilbertson, Chhaya Patel, Shuvro De, Wendy Lo, Michael Garcia-Roig, Thomas M. Austin

**Affiliations:** 1 Department of Pediatric Anesthesiology, Children’s Healthcare of Atlanta, Emory University School of Medicine, 1405 Clifton Road NE, Tower 1 Floor 3, Atlanta, GA 30322, USA; cpatel2@emory.edu (C.P.); wendy.lo@choa.org (W.L.); thomas.austin@emory.edu (T.M.A.); 2 Department of Pediatrics, Children’s Healthcare of Atlanta, Emory University School of Medicine, Atlanta, GA 30322, USA; 3 Department of Pediatric Urology, Children’s Healthcare of Atlanta, Emory University School of Medicine, Atlanta, GA 30322, USA; sde2@emory.edu (S.D.); mgarciaroig@emory.edu (M.G.-R.)

**Keywords:** circumcision, opioid-free anesthesia, pediatric urologic surgery, pediatric anesthesia, pediatric ambulatory surgery

## Abstract

Circumcision is one of the most common urologic procedures performed at pediatric ambulatory centers. Emerging data on the short- and long-term effects of perioperative opioid administration has highlighted the importance of an opioid-free anesthetic regimen. We sought to evaluate the effectiveness of an opioid-free anesthetic in pediatric circumcision and its correlation with ambulatory surgery center efficiency. Patients, 3 years of age and younger, who underwent circumcision or circumcision revision by two surgeons pre and post introduction of an opioid-free anesthetic fast-track regimen at an outpatient surgical center were included. There were 100 patients included in this analysis, with 50 patients in each cohort. On univariate analysis, fast-tracking was associated with a decrease in median combined in-room and post-anesthesia care unit times (102.5 vs. 129.0 min, *p*-value < 0.001). This difference continued after multivariable analysis with an adjusted median combined in-room and post-anesthesia care unit time difference of −15.6 min (95% CI −34.2 to −12.7 min, *p*-value 0.018). In addition, the fast-track cohort received less intraoperative morphine equivalents without an increase in post-operative analgesic administration or change in postoperative questionnaire score. This demonstrates that opioid-free anesthesia may be used effectively in pediatric circumcision while also allowing for significant time savings for surgical centers.

## 1. Introduction

Circumcision is one of the most commonly performed urologic procedures in pediatrics. However, there remains large variability in anesthetic and analgesic techniques for this relatively quick procedure. Most perioperative techniques involve the use of opioids in combination with regional as well as general anesthesia. The national opioid epidemic has shed light on the role anesthesiologists and perioperative opioid administration are contributing to this crisis [1,2]. Among adults, it has been recognized that perioperative opioid use is a risk factor for future opioid misuse [3]. Newer data highlights the increasing trend in opioid dependence among children [4].

Opioid-free anesthesia (OFA) is a multimodal technique which permits for a good quality of anesthesia and analgesia while obviating the need for opioids. In adults, studies have shown that OFA allows for quality pain control while reducing postoperative nausea and vomiting (PONV), decreasing length of stay, and lessening opioid related adverse events [5]. While studies have examined the efficacy of OFA in adult surgical populations, the literature is scarce in pediatric patients. The benefits of OFA may be more substantial in this subset, as opioid induced respiratory depression is of even greater concern in the pediatric population. While one recent review evaluated the feasibility of implementing an OFA regimen in pediatric surgery, there has been insufficient data on the impact of OFA on pediatric ambulatory center efficiency [6].

The institution of a fast-track OFA should theoretically reduce postoperative recovery time, which is a significant indicator of ambulatory surgical center efficiency. This time savings results in the potential for additional patient capacity. The operating room is the fundamental core of a hospital system and maximizing efficiency has important implications for productivity, patient satisfaction, and medical team morale [7].

After implementing an OFA fast-track technique for circumcisions at our free standing pediatric ambulatory surgery center, we sought to evaluate if this practice would result in equivalent analgesia while evaluating its effects on intraoperative and postoperative times. We hypothesized that utilization of this OFA protocol would decrease perioperative time without an increase in adverse events.

## 2. Methods

Prior to data extraction, approval was granted by the Children’s Healthcare of Atlanta (CHOA) Investigational Review Board (IRB #00000913). Inclusion criteria for this analysis consisted of patients who underwent circumcision or circumcision revision by two urologists from 7 January 2020 to 29 December 2020 at CHOA Satellite Boulevard Surgery Center and were 3 years of age or younger. Patients with incomplete medical records or allergies to ibuprofen, acetaminophen, or local anesthetic were excluded from the analysis. Additionally, patients who underwent any additional concurrent procedures such as chordee repair, phalloplasty, lysis of penile adhesions, or urethral meatoplasty were excluded.

The pre-implementation subset consisted of the 50 patients who met the inclusion criteria prior to the initiation of our fast-track OFA regimen. There was no standardized anesthetic prior to introduction of the OFA protocol, but the urologists consistently performed a circumferential dorsal penile nerve block (DPNB). The post-implementation subset consisted of the 50 patients who met the inclusion criteria upon initiating our OFA protocol. Our fast-track OFA technique was standardized for all patients (Table 1). It consisted of premedication with oral acetaminophen 15 mg/kg. A benzodiazepine was not administered preoperatively for anxiolysis as parental present induction is routinely performed at our surgical center. Intraoperatively, patients received 1 mg/kg of intramuscular (IM) ketorolac and 1 mcg/kg of intranasal (IN) dexmedetomidine immediately following inhalational induction with nitrous oxide and sevoflurane. Standard ASA monitors were utilized, including pulse oximeter, electrocardiogram, automated blood pressure cuff, skin temperature probe, end-tidal carbon dioxide, and oxygen/anesthetic gas analyzer. A DPNB was then performed by the urologist using 0.25% bupivacaine 1 mL/kg up to a maximum of 10 mL. Anesthesia was maintained via mask ventilation with sevoflurane and placement of a peripheral intravenous catheter (PIV) was deferred. Upon completion of the procedure, OFA patients were fast-tracked to phase 2 of recovery. Patients receiving opioids recovered in the phase 1 post-anesthesia care unit (PACU) prior to being transferred to phase 2. Patients were discharged home on the same post-operative pain medications pre- and post-intervention.

Per institutional policy, minimum length of stay for circumcisions is sixty minutes after completion of procedure, which was a consistent policy pre- and post-implementation. In-room time was defined as anesthesia start to the time they were moved out of the operating room (OOR), as documented by the circulator. PACU time was defined as the time recovery started, either in phase 1 or 2 depending on if fast-track was utilized, to the time the patient left the surgery center. The questionnaire (Q) score was developed from the standardized nurse post-operative phone call that was utilized pre- and post-implementation. It consists of multiple questions to evaluate parental satisfaction and patient condition post-operatively on a continuum score from 0 to 3. A score of 0 signifies parental dissatisfaction and poor patient state post-operatively, indicated by unsatisfactory pain control, nausea, and vomiting. A score of 3 signifies parental satisfaction and patient pain well controlled without nausea and vomiting.

## 3. Statistical Analysis

Fisher’s Exact Test or the Wilcoxon Rank Sum Test was used to determine univariate differences between the two cohorts based on the distribution of the data. Since this was a before–after study design, interrupted time series analyses were utilized to determine differences between in-room and PACU times with the interruption occurring at the implementation of fast-tracking (29 September 2020) [8]. Since these response variables were positively skewed, multivariable quantile regression models were employed to adjust for potential confounding variables including background temporal trends. Variables included in the models were determined a priori and included age, race/ethnicity (i.e., non-Hispanic White vs. others), ASA classification, surgeon, surgical procedure (i.e., circumcision vs. circumcision revision), intervention (i.e., OFA vs. pre-intervention), pre-intervention time, and post-intervention time. To account for the effects of potential temporal correlation on the regression models, standard errors for each independent variable were created through bootstrapping and confidence intervals (CIs) for the regression coefficients were generated through the Koenker method [9]. All *p*-values were two-sided with values less than 0.05 considered statistically significant. R statistical software (version 4.0.3) was used for this investigation.

For power analysis, a median difference of 15 min for combined in-room and PACU times between the two cohorts was determined to be clinically significant. From preliminary data, median and standard deviation pre-fast-track combined in-room and PACU time was found to be approximately 100 and 15 min, respectively. In order to show a median difference of 15 min (115 vs. 100 min) with standard deviations of 15 min for each group, alpha error of 0.05, and beta error of 0.1, 48 patients were required in each cohort [10].

## 4. Results

**Figure 1:**
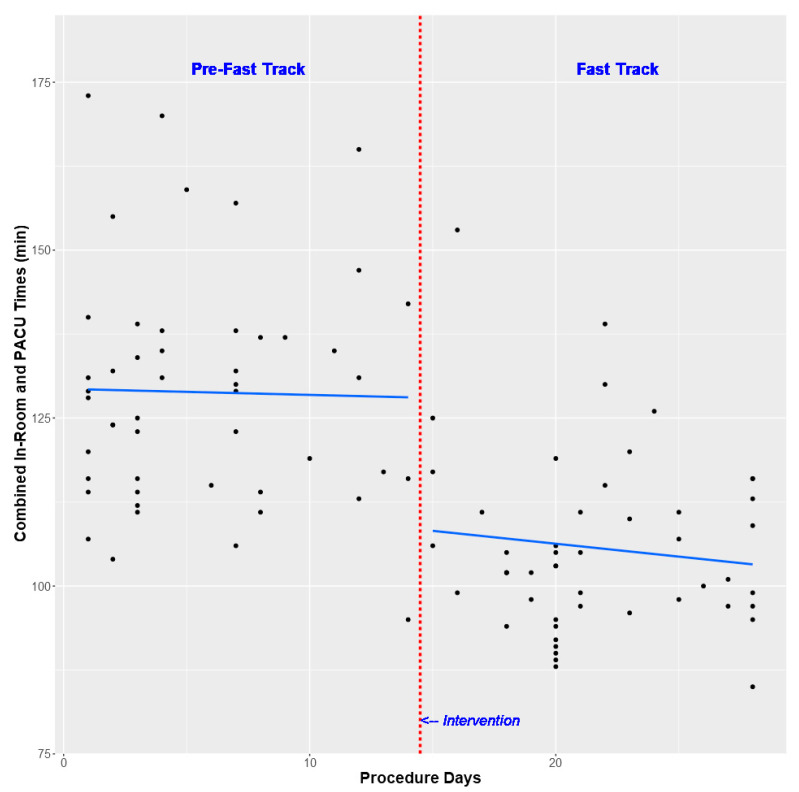
Scatter plot with superimposed segmented simple median regression line with break point at time of fast track implementation (procedure day 15). The Y variable is combined in-room and PACU times for each procedure while the X variable is the sequential days that circumcisions and/or circumcision revisions were performed over the entire study period. Based on this graph, there appears to be a stepped decrease in combined time that corresponds to the implementation of fast tracking.

There were 100 patients included in this analysis with 50 patients in each cohort. Median [interquartile range (IQR)] age was 12.0 [8.0, 20.0] months with the majority of patients being American Society of Anesthesiologists (ASA) physical status 1 or 2 and non-White (Table 2). On univariate analysis, fast tracking was associated with a decrease in median combined in-room and PACU times (102.5 vs. 129.0 min, *p*-value < 0.001, Table 3 and Figure 1). This difference continued after multivariable analysis with an adjusted median combined in-room and PACU time difference of −15.6 min (95% CI −34.2 to −12.7 min, *p*-value 0.018, Table 4). This time savings was comprised of both decreased in-room times (adjusted median difference −9.3 min, 95% CI −14.6 to −4.7, *p*-value 0.003, Table 4) and decreased PACU times (adjusted media difference −15.0, 95% CI −26.1 to −2.9, *p*-value 0.026, Table 4). In addition, the OFA cohort received no opioids without an increase in PACU analgesic administration or change in postoperative Q scores (Table 3). Lastly, there were no differences in perioperative respiratory events between the two groups (Table 3).

## 5. Discussion

This study demonstrates an effective and efficient way of providing OFA for pediatric patients undergoing circumcision. Historically, our institution followed a similar model to many practices of performing circumcisions under general anesthesia with a supplemental DPNB placed by the surgeon. With this technique, we administered an average of 0.15 mg/kg morphine equivalents throughout the perioperative period. In order to apply our OFA fast-track model, we decided on the use of preoperative oral acetaminophen in addition to intraoperative intranasal dexmedetomidine and intramuscular ketorolac for a multimodal approach. The surgeons continued to perform the DPNB prior to the start of the procedure, as was previously done. After implementing the OFA protocol, no patients received preoperative opioids nor required opioids during the intraoperative or postoperative phases. Postoperative phone calls to parents showed no increase in pain experienced after discharge and equivalent parental satisfaction, as represented by the Q score. In addition, we had no significant increase in perioperative respiratory complications or unplanned admissions as a result of the OFA fast-track protocol.

The implementation of OFA has started to gain immense popularity in the adult population. However, there is scarce literature regarding its use in pediatrics, especially in common urologic procedures such as circumcisions. Recent studies have shown that the unexpected admission rate for pediatric circumcision ranges from 2–3%, likely due to uncontrolled pain and PONV [11]. In another pediatric study, over half of parents reported their child experienced moderate to severe pain postoperatively [12]. There continues to be debate as to which anesthetic regimen is best for these procedures. Everything from purely local anesthetic under spinal anesthesia to general with caudal or DPNB has been evaluated. In a review of pediatric surgeons using multi-criteria decision making models, the preferred choice of anesthesia for circumcision was general anesthesia with a DPNB [13]. While DPNBs have been found to be successful in reducing pain, anesthesiologists typically supplement with intravenous opioids to provide improved analgesia [14,15].

In light of the recent data demonstrating the benefit of OFA, we wanted to target an applicable population at our ambulatory surgery center. Our goal was to develop an anesthetic that provided appropriate surgical conditions and satisfactory pain control while mitigating the risk of opioid induced effects. The importance of reducing opioid administration, specifically in this pediatric population, continues to be emphasized by ongoing research [16]. While the effect of respiratory depression is obviously of critical importance in this young age group receiving ambulatory surgery, there is now more confirmation of the potential of long-term effects from the use of perioperative opioids. The possibility of postoperative hyperalgesia and new persistent opioid use from perioperative opioid administration is evident in the literature [17,18].

Upon executing this technique, we wanted to ensure that we did not sacrifice our essential value of efficiency in the ambulatory setting. While there is limited literature of the use of OFA in pediatric anesthesia, there is even scarcer data of its effects on perioperative productivity. The use of dexmedetomidine as an effective analgesic adjunct has often been reported, however the undesirable prolonged wake-up and discharge associated with its use was of concern for us [19]. The results of this analysis show that we actually improved efficiency by applying our OFA protocol. By using an OFA system, we were able to implement a fast-track technique in which we were able to bypass phase 1 of recovery by eliminating the use of opioids intraoperatively. Parental presence during the postoperative period has increasingly been found to be beneficial, especially in the incidence of emergence delirium [20,21].

While patients had to remain in recovery for a one-hour observation period to ensure no extensive bleeding, the postoperative recovery phase was substantially reduced from 85 to 62 min. Importantly, we found that our OFA fast-track protocol also resulted in a significant decrease in in-room time as a result of not placing a PIV and avoiding airway manipulation. As we allow for one-hour clear fasting times and we have an average surgical procedure time of 22 min, we were comfortable not placing a PIV for hydration status. We improved from an average of 41.5 min in-room pre-intervention to 37 min post- with these key changes. After adjusting for cofounders, this resulted in an overall combined decrease of in-room and PACU time of 15.6 min. In a busy ambulatory setting, in which we perform 20 of these cases in a single day, this decrease in time leads to a meaningful savings of 312 min in just one day.

During this time, we experienced two episodes of laryngospasm resolved with positive pressure and one episode of laryngospasm on induction requiring IM succinylcholine. Because we were very cognizant during implementation of our OFA fast-track protocol, we documented any event that occurred. As we have a paper charting system, which results in inconsistencies based upon personnel charting such events, we may have had undocumented laryngospasms resolved with positive pressure that were not captured pre-intervention. We did not experience any intraoperative respiratory depression or obstructive episodes that necessitated PIV placement or intubation after introduction of our protocol.

## 6. Limitations

There are limitations intrinsic to this study as the review was retrospectively performed. Due to the inherent potential for bias from the retrospective nature of this review, we sought to eliminate multiple extraneous factors in our analysis. Prior to the institution of the OFA protocol, there was no standardized anesthetic for circumcisions at our institution. As this allowed for high variability in provider practices, we assessed numerous elements that may contribute to potential bias. We then attempted to account for this inconsistency by including multiple of these confounders in our analysis. We also incorporated the type of surgical procedure, circumcision versus circumcision revision, as we know there are fundamentally different average procedural lengths between the two groups in our practice. Individual charting practices also account for variability within our system. For this reason, we used anesthesia start to OOR as documented by the circulator as our measure of in-room time.

Restraints also exist in our documentation of postoperative pain. While nurses predominantly use the Face, Legs, Activity, Cry, Consolability (FLACC) pain scale, the timing of pain evaluations and the occasional inconsistency of documentation leads to difficulty in using this as an adequate indicator of postoperative pain. For this reason, we used postoperative pain medication requirements and postoperative parent phone call questionnaires to provide more consistency and accuracy.

Applicability of this practice for older patients still needs to be delineated. We chose an age group of 3 years and younger because of the ability to adequately perform a DPNB in this age group, as our surgeons do not inject more than a total of 10 mL of bupivacaine for this block. Further evaluation in older children may show that a completely OFA regimen is not as favorable as in the population we evaluated, but the same protocol may potentially lead to a significant decrease in the overall morphine equivalents administered.

While this technique was highly successful at our institution, there are many factors that allowed for this success. The proficiency of the whole operating room staff from surgeons to circulators to anesthesia team plays an essential role in the feasibility of applying this technique. We perform these procedures frequently and batch them on the same day, so that the operating room staff can be as prepared and efficient as possible. This is also much easier to implement at a free standing pediatric ambulatory center such as ours that is well versed in fast, outpatient procedures in children.

## 7. Conclusions

This study shows that the use of OFA fast-track anesthesia for pediatric circumcision may provide an effective anesthetic while affording improved efficiency. By combining a DPNB with a multimodal technique utilizing acetaminophen, ketorolac, and dexmedetomidine, we were able to completely eliminate perioperative opioid use. The utilization of this protocol produces a substantial decrease in in-room and PACU recovery time. This may result in significant time savings, which is essential for a fast-paced surgical center.

## Figures and Tables

**Table 1:** Opioid-Free Fast Track Protocol.

Premedication with oral acetaminophen (15 mg/kg)
Inhalation anesthetic induction with nitrous oxide and sevoflurane
Intramuscular ketorolac (1 mg/kg) immediately after induction
Intranasal dexmedetomidine (1 mcg/kg) immediately after induction
Dorsal penile nerve block with 0.25% bupivacaine (1 cc/kg up to a maximum of 10 cc)
No peripheral intravenous catheter
Maintenance anesthetic with sevoflurane by mask
Phase II recovery postoperatively with immediate parental presence

**Table 2:** Patient Demographics and Preoperative Characteristics.

	Fast Track (*n* = 50)	Pre-Fast Track (*n* = 50)
**Age (Months)**	11.5 (7.0, 19.8)	13.5 (9.0, 20.0)
**Race/Ethnicity (Non-Hispanic White)**	20 (40.0%)	16 (32.0%)
**ASA Classification**		
1	46 (92.0%)	39 (78.0%)
2	4 (8.0%)	10 (20.0%)
3	0 (0.0%)	1 (2.0%)
**Surgeon**		
1	26 (52.0%)	22 (44.0%)
2	24 (48.0%)	28 (56.0%)
**Procedure**		
Circumcision	32 (64.0%)	40 (80.0%)
Circumcision Revision	18 (36.0%)	10 (20.0%)

Abbreviation: ASA = American Society of Anesthesiologists. Data presented as count (percentage) or median [25% percentile, 75% percentile].

**Table 3:** Key Perioperative Variables.

	Fast Track (*n* = 50)	Pre-Fast Track (*n* = 50)	*p*-Value *
**Combined In-Room and PACU Time (min)**	102.5 (97.0, 111.0)	129.0 (116.0, 137.8)	<0.001
**In-Room Time (min)**	37.0 (31.0, 42.0)	41.5 (34.3, 50.8)	0.002
**PACU Time (min)**	62.0 (60.0, 76.0)	85.0 (75.3, 91.0)	<0.001
**Intraoperative Morphine Equivalents (mg/kg)**	0 (0, 0)	0.15 (0.10, 0.19)	<0.001
**Postoperative Analgesic Administration (Yes)**	0 (0.0%)	3 (6.0%)	0.24
**Postoperative Q Score (0–3)**	3 (3, 3)	3 (3, 3)	0.24
**Perioperative Respiratory Complications (Yes)**	3 (6.0%)	0 (0.0%)	0.24

Abbreviations: PACU = Post-anesthesia Care Unit, Q = Quality. Data presented as count (percentage) or median [25% percentile, 75% percentile]. * Based on Wilcoxon Rank Sum Test or Fisher’s Exact Test based on the distribution of the data. *p*-value < 0.05 considered statistically significant.

**Table 4:** Association between Fast Track and In-Room/PACU Time.

		^§^ Unadjusted			^¶^ Adjusted		
Outcome	Levels	Coefficient	95% CI	*p*-Value	Coefficient	95% CI	*p*-Value
**Combined In-Room and PACU Time (min)**	Fast Track vs. Pre-Fast Track (Referent)	−23.0	−29.0 to −17.0	<0.001	−15.6	−34.2 to −12.7	0.018
**In-Room Time (min)**	Fast Track vs. Pre-Fast Track (Referent)	−6.0	−9.0 to −2.0	0.002	−9.3	−14.6 to −4.7	0.003
**PACU Time (min)**	Fast Track vs. Pre-Fast Track (Referent)	−17.0	−22.0 to −12.0	<0.001	−15.0	−26.1 to −2.9	0.026

CI = Confidence Interval; PACU = Post-anesthesia Care Unit. ^**§**^ Based on the Wilcoxon Rank Sum Test. *p*-values < 0.05 considered statistically significant. **^¶^** Based on interrupted times series analysis using multivariable median regression modelling to adjust for age, ASA classification, race/ethnicity, surgeon, surgical procedure, and pre- and post-intervention temporal trends. *p*-values < 0.05 considered statistically significant.

## Data Availability

The data that support the findings of this study are available from the corresponding author upon reasonable request.

## Abbreviations

OFA	Opioid-free Anesthesia
PONV	Post-operative Nausea and Vomiting
CHOA	Children’s Healthcare of Atlanta
DPNB	Dorsal Penile Nerve Block
IM	Intramuscular
IN	Intranasal
PIV	Peripheral Intravenous Line
PACU	Post-Anesthesia Care Unit
OOR	Out of OR
Q	Questionnaire
CI	Confidence Interval
IQR	Interquartile Range
ASA	American Society of Anesthesiologists
FLACC	Face, Legs, Activity, Cry, Consolability
